# A novel protein encoded by a circular RNA circPPP1R12A promotes tumor pathogenesis and metastasis of colon cancer via Hippo-YAP signaling

**DOI:** 10.1186/s12943-019-1010-6

**Published:** 2019-03-29

**Authors:** Xiao Zheng, Lujun Chen, You Zhou, Qi Wang, Zhuojun Zheng, Bin Xu, Chen Wu, Qi Zhou, Wenwei Hu, Changping Wu, Jingting Jiang

**Affiliations:** 1 Department of Tumor Biological Treatment, the Third Affiliated Hospital of Soochow University, Changzhou, 213003 People’s Republic of China; 2 Jiangsu Engineering Research Center for Tumor Immunotherapy, Changzhou, 213003 People’s Republic of China; 3 Institute of Cell Therapy, Soochow University, Changzhou, 213003 People’s Republic of China; 4 Department of Hematology, the Third Affiliated Hospital of Soochow University, Changzhou, 213003 People’s Republic of China; 5 Department of Oncology, the Third Affiliated Hospital of Soochow University, Changzhou, 213003 People’s Republic of China

**Keywords:** Colon cancer, Circular RNA (circRNA), Protein coding, Proliferation, Metastasis

## Abstract

**Background:**

It has been well established that circular RNAs (circRNAs) play an important regulatory role during tumor progression. Recent studies have indicated that even though circRNAs generally regulate gene expression through miRNA sponges, they may encode small peptides in tumor pathogenesis. However, it remains largely unexplored whether circRNAs are involved in the tumorigenesis of colon cancer (CC).

**Methods:**

The expression profiles of circRNAs in CC tissues were assessed by circRNA microarray. Quantitative real-time PCR, RNase R digestion assay and tissue microarray were used to confirm the existence and expression pattern of circPPP1R12A. The subcellular distribution of circPPP1R12A was analyzed by nuclear mass separation assay and fluorescence in situ hybridization (FISH). SDS-PAGE and LC/MS were employed to evaluate the protein-coding ability of circPPP1R12A. CC cells were stably transfected with lentivirus approach, and cell proliferation, migration and invasion, as well as tumorigenesis and metastasis in nude mice were assessed to clarify the functional roles of circPPP1R12A and its encoded protein circPPP1R12A-73aa. RNA-sequencing and Western blotting analysis were furthered employed to identify the critical signaling pathway regulated by circPPP1R12A-73aa.

**Results:**

We firstly screened the expression profiles of human circRNAs in CC tissues and found that the expression of hsa_circ_0000423 (termed as circPPP1R12A) was significantly increased in CC tissues. We also found that circPPP1R12A was mostly localized in the cytoplasm of CC cells. Kaplan–Meier analysis showed that patients with higher levels of circPPP1R12A had a significantly shorter overall survival. By gain- and loss-of-function approaches, the results suggested that circPPP1R12A played a critical role in proliferation, migration and invasion of CC cells. Furthermore, we showed that circPPP1R12A carried an open reading frame (ORF), which encoded a functional protein (termed as circPPP1R12A-73aa). Next, we found that PPP1R12A-C, not circPPP1R12A, promoted the proliferation, migration and invasion abilities of CC in vitro and in vivo. Finally, we identified that circPPP1R12A-73aa promoted the growth and metastasis of CC via activating Hippo-YAP signaling pathway. In addition, the YAP specific inhibitor Peptide 17 dramatically alleviated the promotive effect of circPPP1R12A-73aa on CC cells.

**Conclusions:**

In the present study, we illustrated the coding-potential of circRNA circPPP1R12A in the progression of CC. Moreover, we identified that circPPP1R12A-73aa promoted the tumor pathogenesis and metastasis of CC via activating Hippo-YAP signaling pathway. Our findings might provide valuable insights into the development of novel potential therapeutic targets for CC.

**Electronic supplementary material:**

The online version of this article (10.1186/s12943-019-1010-6) contains supplementary material, which is available to authorized users.

## Introduction

Colon cancer (CC) is a common type of malignant tumor worldwide, which is the third frequently detected cancer and the fourth leading cause of cancer-related death [1]. Although the therapeutic approaches for CC have been greatly improved, more than half of CC patients ultimately die. Therefore, it is urgently necessary to explore other unknown mechanisms underlying the progression of CC [2–4].

With the rapid development of next-generation sequencing technologies, more and more previously unknown transcripts have been identified. Non-coding RNAs (ncRNAs), as most of above-mentioned transcripts, can not be translated into proteins due to the lack of obvious open reading frames (ORFs) in organisms [5–8]. Circular RNAs (circRNAs), a class of circularly configured RNA molecules consisting of an upstream splice acceptor and a downstream splice donor [9], have been recently reported to be widely spread in eukaryotes. There are three types of circRNAs according to their origins, including exonic, intronic and intergenic regions. The exonic circRNAs (originated from exonic sequences) typically reside in the cytoplasm, while intronic circRNAs (intron-retaining circRNAs) mostly remain in nuclei [10]. The exonic circRNAs, the end-products of splicing, are the most studied circRNAs [11]. Due to their abundance and stability, it is well established that certain circRNAs can widely regulate biological activities by functioning as ceRNA or regulators of RNA binding protein in modulating protein-gene transcription [12–15]. Recently, several circRNAs have been found to encode proteins [16–18]. However, it remains unclear whether there are protein-coding circRNAs involved in CC tumorigenesis, and their functional products are still elusive.

In the present study, we identified an up-regulated circRNA hsa_circ_0000423 (termed as circPPP1R12A) in CC tissues. Silencing of circPPP1R12A by siRNAs suppressed the proliferation ability of CC cells, while the migration and invasion abilities of CC cells remained unaffected. Moreover, a 73-amino acid (aa) protein encoded by circPPP1R12A was identified by overlapping genetic codes and proteomics. The in vitro and in vivo data showed that when the protein-coding ability of circPPP1R12A was abolished by mutating the start codon, its promotive effect on cell proliferation was reversed. Moreover, we identified that circPPP1R12A-73aa promoted the growth and metastasis of CC via activating Hippo-YAP signaling pathway. Collectively, a protein encoded by circRNA circPPP1R12A contributed to the rapid proliferation of CC cells.

## Materials and methods

### Patients and tissue samples

A total of 20 paired human CC and adjacent non-tumor tissues were collected between 2014 and 2017 at the Third Affiliated Hospital of Soochow University. This study was approved by the Ethical Committee of the Third Affiliated Hospital of Soochow University, and the written informed consent was provided by each participant prior to surgery.

### Expression profile analysis of circRNAs

The circRNA microarray (Arraystar Human circRNAs chip, ArrayStar) consisting of more than 5000 probes specific to splicing sites of human circRNAs was used. After hybridization, 20 pairs of CC samples (tumor tissues and paired adjacent non-tumor tissues) were examined using the circRNA micro-array provided by Kangcheng Bio-Tech Inc.

### Cell lines and cell culture

Human CC cell lines HT-29, HCT-116, SW480, SW620, LoVo, SW48, DLD-1, Caco2 and HCT-15) and normal human colon mucosal epithelial cell line NCM460 were obtained from Chinese Academy of Sciences, Shanghai Institutes for Biological Sciences. All these cell lines were maintained in DMEM supplemented with 10% FBS under standard culture conditions (5% CO_2_, 37 °C).

### RNA extraction, gDNA extraction, and quantitative real-time PCR analysis

RNA extraction and quantitative real-time PCR were performed as previously described [19]. Genomic DNA (gDNA) was extracted from tissues using PureLink Genomic DNA Mini Kit according to the manufacturer’s instructions (Thermo Fisher Scientific, K182001). The real-time PCR was conducted using the ABI Vii7 system (Applied Biosystems, USA), and SYBR Green was used as a DNA-specific fluorescent dye. Human GAPDH was selected as a housekeeping gene. Primers were synthesized as follows, the human circPPP1R12A forward primer: 5′-ACAGCAGCAGGCTAGAAAAG-3′, and reverse primer: 5′-TGTCCTAAGCAGGAAAAACA-3′; GAPDH forward: 5′-TGACTTCAACAGCGACACCCA-3′, and GAPDH reverse: 5′-CACCCTGTTGCTGTAGCCAAA-3′. Relative gene expression was calculated by the comparative CT method (ΔΔCT), where the fold enrichment was determined as: 2^-[ΔCT (sample) − ΔCT (calibrator)]^.

### RNA in situ hybridization (ISH) and fluorescence in situ hybridization (FISH)

ISH and FISH were carried out to detect the circPPP1R12A expression in colon tissue microarray (Shanghai Outdo Biotech, Shanghai, China) using digoxigenin- or FITC-labeled probe as previously reported [20]. After stained with an anti-digoxin or anti-FITC mAb (Roche Applied Science), the sections were incubated in the presence or absence of NBT/BCIP in the dark, mounted and examined under microscope.

### Plasmids, siRNAs and cell transfection

Human circPPP1R12A over-expressing vector (Lv-circPPP1R12A) and the control plasmid (pLVX-IRES-GFP) were purchased from Genelily Biotechnology Company (Shanghai, China). The expression of circPPP1R12A was transiently silenced by small interference RNAs (siRNAs) specific to human circPPP1R12A, which were generated by GenePharma (GenePharma Corporation, Shanghai, China). The experiment was divided into several groups as follows: si-NC (transfected with siRNA NC vector) group, si-circPPP1R12A (transfected with circPPP1R12A siRNA) group, OE-NC (transfected with over-expression NC vector) group and OE-circPPP1R12A (transfected with circPPP1R12A over-expression vector) group. The above-mentioned vectors were transfected by Lipofectamine® 3000 transfection reagent as previously reported. After transfection for 48 h, the transfection efficiency of cells in each group was assessed by real-time PCR analysis.

### Cell proliferation, colony formation and cell cycle assays

Cell growth was monitored by CCK8 assay as previously reported [21]. Colony formation assays were performed to indicate the cloning capability of CC cells. For cell cycle assay, subG1, S and G2 peaks were detected from the propidium iodide-stained CC cells by flow cytometry (BD FACS Canto II, BD Bioscience, NJ, USA) and analyzed using the Modfit software.

### Migration and invasion assay

The cell migration ability was assessed using wound-healing assay as previously reported. The invasion assay was analyzed by transwell chamber with 8-μm pores (BD Falcon, Franklin Lakes, NJ, USA).

### Western blotting analysis

Western blotting analysis was performed as previously described [19] with antibodies against flag and GAPDH (Abcam, Cambridge, MA, USA). GAPDH was used as an endogenous control to normalize the protein loading.

### Analysis of peptide patterns by LC-MS/MS

The protein identification by LC-MS/MS was performed according to previously reported method [22]. Briefly, equal amounts of proteins (50 μg) were subjected to 12% SDS-PAGE. The protein bands near 10 kDa were excised according to size and chopped into 1 mm^3^ pieces. Finally, peptide mixtures were extracted from the gels and dried prior to LC-MS/MS analysis.

### Statistical analysis

Statistical analyses were completed using the SPSS version 16.0 (SPSS Inc., Chicago, IL, USA). Survival analysis was carried out using the Kaplan–Meier method, and the log-rank test was used to compare the survival curves. Other data were assessed with Student’s two-tailed t-test by GraphPad Prism 5.0 software package (GraphPad Software, Inc., San Diego, USA). A *P* value < 0.05 was considered as statistically significant.

## Results

### Expression profiles and screening of circRNAs in CC tissues and cells

**Fig. 1:**
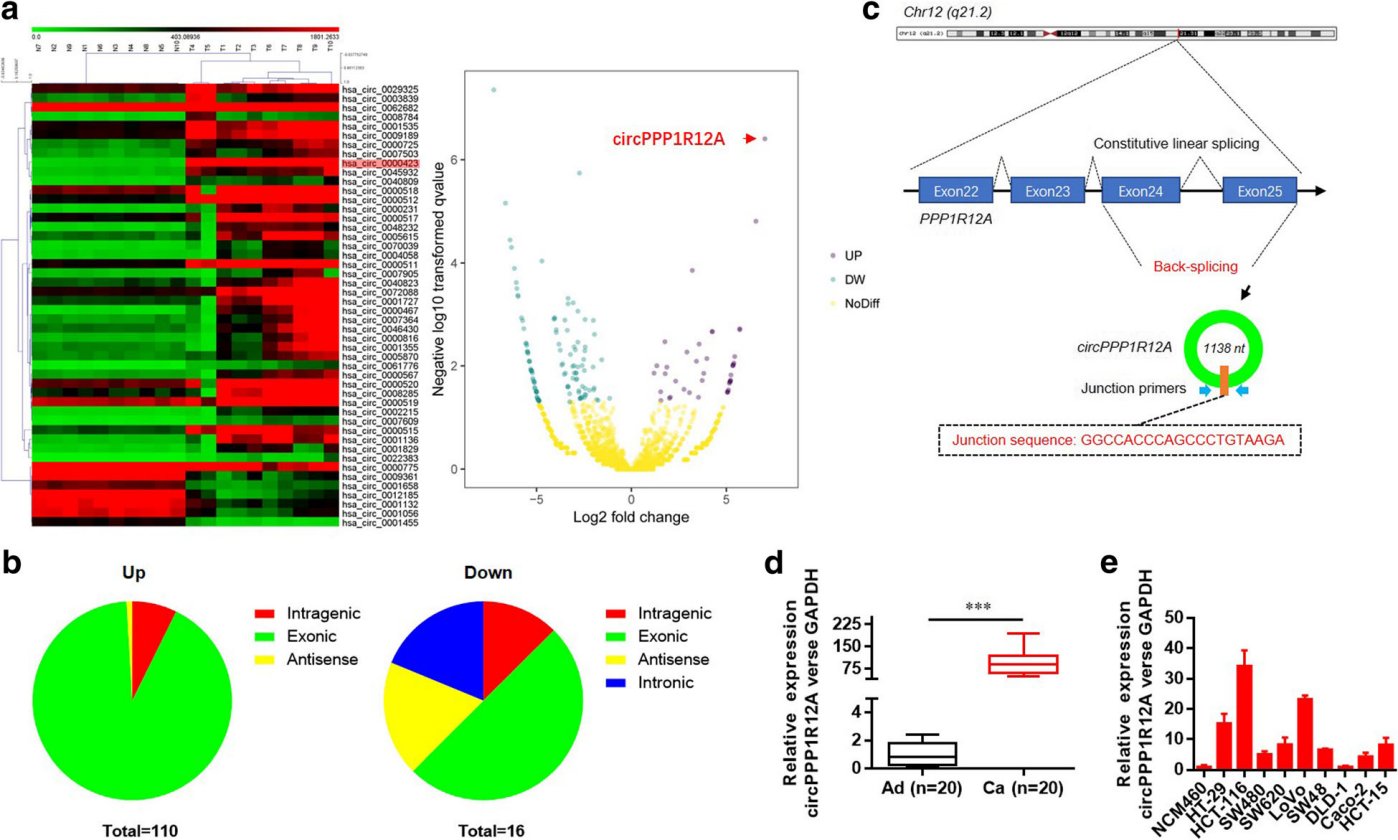
CircRNA expression profile in CC and characterization of circPPP1R12A. **a** Heatmap of the differentially expressed circRNAs in 10 pairs of human CC tissues and matched non-tumor tissues. **b** Classification of dysregulated circRNAs. **c** CircPPP1R12A was back-spliced by exons 24 and 25 of PPP1R12A gene located at 12q21.2. **d** The expression level of circPPP1R12A in CC and matched non-tumor tissue samples from 20 patients was analyzed by real-time PCR. **e** The expression level of circPPP1R12A in a series of cultured CC cell lines (HT-29, HCT-116, SW480, SW620, LoVo, SW48, DLD-1, Caco2 and HCT-15) was analyzed by real-time PCR. ***P < 0.001

Firstly, circRNA microarray was employed to characterize the expression profiles of circRNAs in paired CC tissues and adjacent non-tumor tissues from 10 patients. A total of 126 circRNAs (*P* < 0.05 and fold change > 1.5) were differentially expressed between the CC tissues and paired adjacent non-tumor tissues. Among the 126 differentially expressed circRNAs, 110 circRNAs were up-regulated, while 16 ones were down-regulated in CC tissues compared with the adjacent non-tumor tissues (Fig. 1 a). Additional file 1: Table S1 lists the detailed information about these dysregulated circRNAs. These circRNAs were mostly located at exonic regions (Fig. 1 b). As the most up-regulated circRNA, hsa_circ_0000423 (termed as circPPP1R12A) was back-spliced of exons 24/25 of PPP1R12A gene located at 12q21.2 (Fig. 1 c). Next, we re-examined the expression of circPPP1R12A in CC and paired non-tumor tissue samples from 20 patients by quantitative real-time PCR to confirm its elevated expression (Fig. 1 d). We further found that the circPPP1R12A expression was consistently and significantly increased in CC tissues compared with the matched controls, while the expression of PPP1R12A (linear transcript of PPP1R12A gene) was comparable in CC tissues and matched controls (Additional file 2: Figure S1a). Moreover, the expression of circPPP1R12A was significantly up-regulated in a series of cultured CC cell lines (HT-29, HCT-116, SW480, SW620, LoVo, SW48, DLD-1, Caco2 and HCT-15) compared with a normal human colon mucosal epithelial cell line NCM460 cells. The highest expression of circPPP1R12A was found in HCT-116 cells, followed by LoVo cells (Fig. 1 e). Therefore, our subsequent experiments focused on the role of circPPP1R12A in CC progression.

### Characterization of the existence and subcellular distribution of circPPP1R12A in CC cells and tissues

**Fig. 2:**
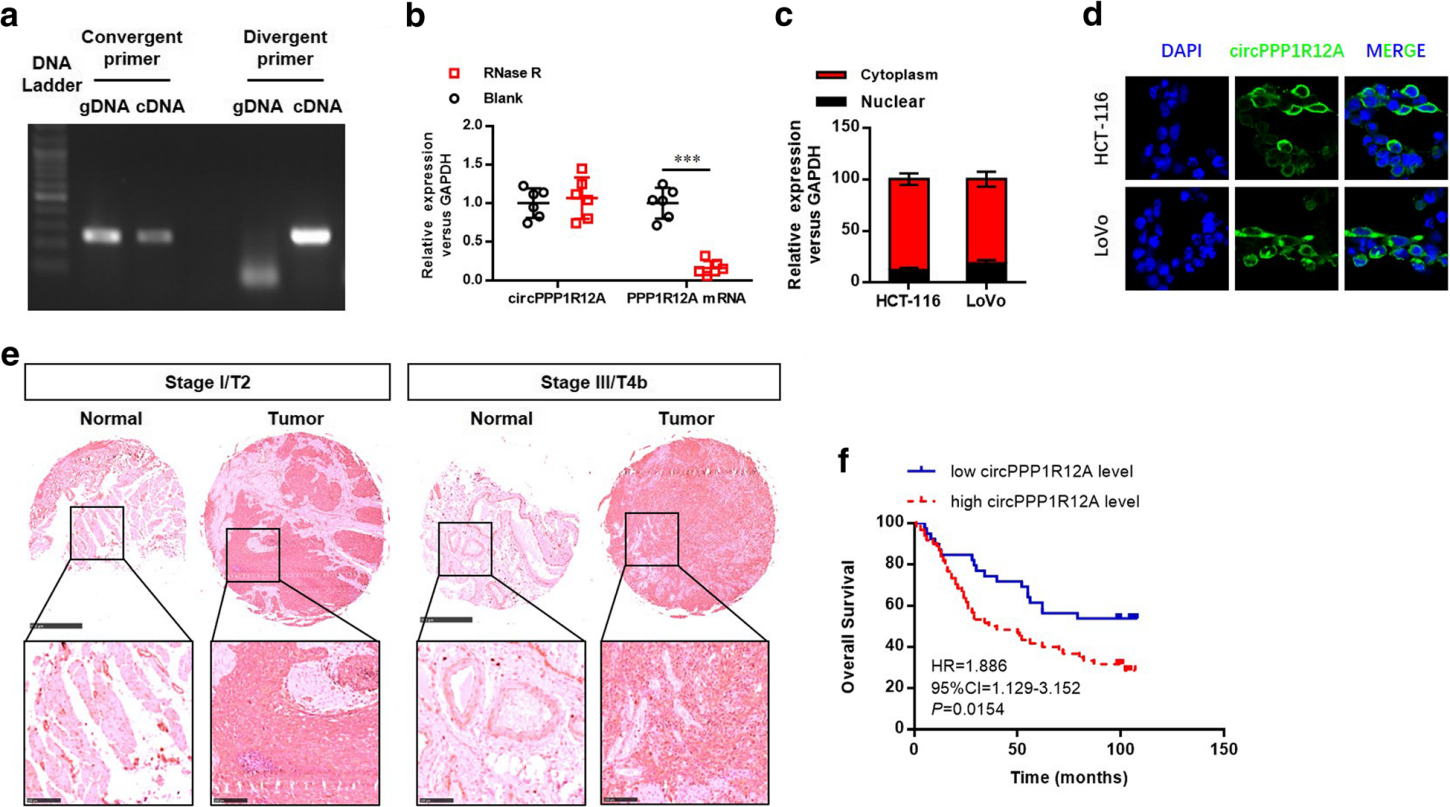
Characterization the existence and subcellular distribution of circPPP1R12A in CC cells and tissues. **a** The divergent primers detected circPPP1R12A in cDNA but not in gDNA. **b** Real-time PCR analysis of circPPP1R12A and linear PPP1R12A mRNA after treatment with RNase R in HCT-116 cells showed that circPPP1R12A was resistant to RNase R treatment. The sub-cellular distribution of circPPP1R12A was mostly present in the cytoplasm by the nuclear mass separation assay (**c**) and FISH (**d**). **e** The level of circPPP1R12A was analyzed by in situ hybridization on CC tissue microarray, showing that circPPP1R12A was up-regulated in CC tissues compared with normal tissues, and such up-regulation was positively correlated with larger tumors and a higher TNM stage. **f** Kaplan–Meier analysis of the correlation between circPPP1R12A expression and overall survival showed that patients with higher levels of circPPP1R12A had a significantly shorter overall survival. ***P < 0.001

In the present study, we designed two sets of primers to characterize circPPP1R12A. One pair (divergent primers) was used to amplify the circular transcripts, while the other pair (convergent primers) was used to detect the linear transcripts. The results suggested that the circular form could be amplified using the convergent primers from both cDNA and gDNA, while it was only amplified from cDNA by divergent primers (Fig. 2 a). To further confirm the existence of circPPP1R12A, the RNase R degradation assay was used to evaluate the resistance of circPPP1R12A to RNase R treatment. Figure 2 b shows that the linear transcripts of PPP1R12A were degraded by RNase R treatment, while such treatment failed to degrade the circular transcripts of circPPP1R12A. Nuclear mass separation assay (Fig. 2 c) and FISH analysis (Fig. 2 d) reveled that over 93% of circPPP1R12A appeared in the cytoplasm of HCT-116 and LoVo cells. We also detected the expression of circPPP1R12A in CC tissues by ISH using TMA consisting of 100 pairs of CC and adjacent non-tumor tissues (Fig. 2e). Table 1 lists the detailed clinical parameters of these patients. Among the clinicopathological variables, pathological stage and circPPP1R12A ISH score were identified as risk factors for predicting overall survival based on univariate analysis, while multivariate analysis with Cox regression model further confirmed that pathological stage III and circPPP1R12A ISH score 3–4 were the independent poor prognostic factors (Table 2). Kaplan–Meier survival curves showed that patients with higher expression of circPPP1R12A had a shorter overall survival [HR = 1.886; 95% confidence interval (CI), 1.129–3.1529; *P* = 0.0154; Fig. 2 f].

**Table 1:** The detailed clinical parameters of the 100 patients

Clinical Parameters	Cases
Gender	
Male	76
Female	24
Age (years)	
< 55	65
≥55	35
Tumor size (cm)	
≤5	46
> 5	54
Pathological stage	
I + II	42
III	58
Pathological type	
Massive	24
Nodular	76
T stage	
I + II	27
III + IV	73

**Table 2:** Univariate and multivariate survival analysis of CC patients from SEER database

Variable	Univariate analysis		Multivariate analysis	
	HR (95% CI)	*P*	HR (95% CI)	*P*
Sex (Male vs. Female)	0.935 (0.543–1.611)	0.809		
Age (> 55 vs. 16–55 years)	1.620 (0.584–4.495)	0.354		
Tumor size (cm) (> 5 vs. 0–5)	1.007 (0.562–1.802)	0.982		
Pathological stage (III vs. I + II)	2.815 (1.520–5.215)	0.001	2.865 (1.521–5.394)	0.001
Pathological type (Massive vs. Nodular)	1.565 (0.907–2.701)	0.108		
circPPP1R12A ISH Score (3–4 vs. 0–2)	2.788 (1.484–5.237)	0.001	2.927 (1.574–5.443)	0.001

Abbreviation: *ISH*, In Situ Hybridization

### CircRNA circPPP1R12A promotes the cell proliferation, but not migration and invasion abilities of CC cells

**Fig. 3:**
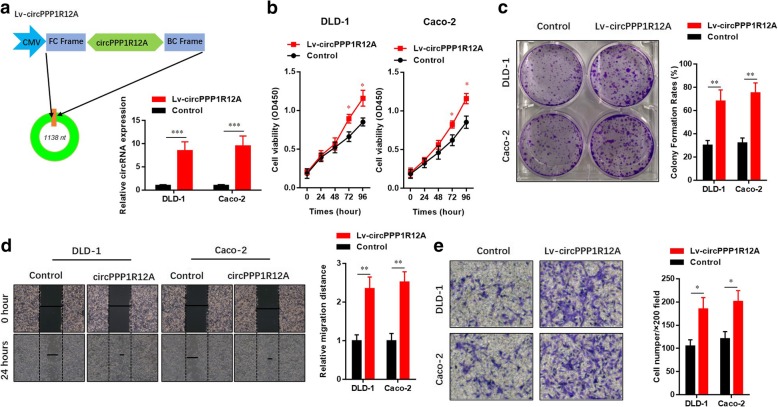
CircRNA circPPP1R12A promotes the cell proliferation, but not migration and invasion abilities of CC cells. **a** Schematic illustration and real-time PCR confirmation showing circPPP1R12A overexpression in DLD-1 and Caco-2 cells. **b** CircPPP1R12A promoted the proliferation of DLD-1 and Caco-2 cells shown by CCK8 assay. **c** CircPPP1R12A promoted the proliferation of DLD-1 and Caco-2 cells shown by colony formation assay. **d** CircPPP1R12A did not affect the migration of DLD-1 and Caco-2 cells shown by wound healing assay. **e** CircPPP1R12A did not affect the invasion of DLD-1 and Caco-2 cells shown by matrigel assay. The data are represented as the means ± SEM; *P < 0.05

As unlimited cell proliferation, migration and invasiveness are hallmarks of malignant tumor, we next explored the role of circPPP1R12A in progression of CC by gain- and loss-of-function approaches. We overexpressed circPPP1R12A by artificial plasmids in two (DLD-1 and Caco-2) CC cell lines with low circPPP1R12A expression, and the overexpression efficiency was confirmed by real-time PCR (Fig. 3 a). CCK8 assay (Fig. 3 b) and colony formation assay (Fig. 3 c) indicated that circPPP1R12A overexpression accelerated the cell proliferation of DLD-1 and Caco-2 cells. Subsequently, we explored the effect of circPPP1R12A on motility of CC cells. Wound-healing assay and invasion assay showed that circPPP1R12A overexpression also enhanced the migration (Fig. 3 d) and invasion (Fig. 3 e) abilities of DLD-1 and Caco-2 cells. Moreover, we overexpressed circPPP1R12A by artificial plasmids in two (HCT-116 and LoVo) CC cell lines with high circPPP1R12A expression. Similarly, circPPP1R12A overexpression accelerated the cell proliferation of HCT-116 and LoVo cells, evidenced by CCK8 assay (Additional file 3: Figure S2a) and colony formation assay (Additional file 3: Figure S2b). However, circPPP1R12A overexpression failed to alert the migration (Additional file 3: Figure S2c) and invasion (Additional file 3: Figure S2d) abilities of DLD and Caco-2 cells in wound-healing assay and invasion assay.

### Silencing of circPPP1R12A suppresses the cell proliferation, migration and invasion abilities of CC cells

**Fig. 4:**
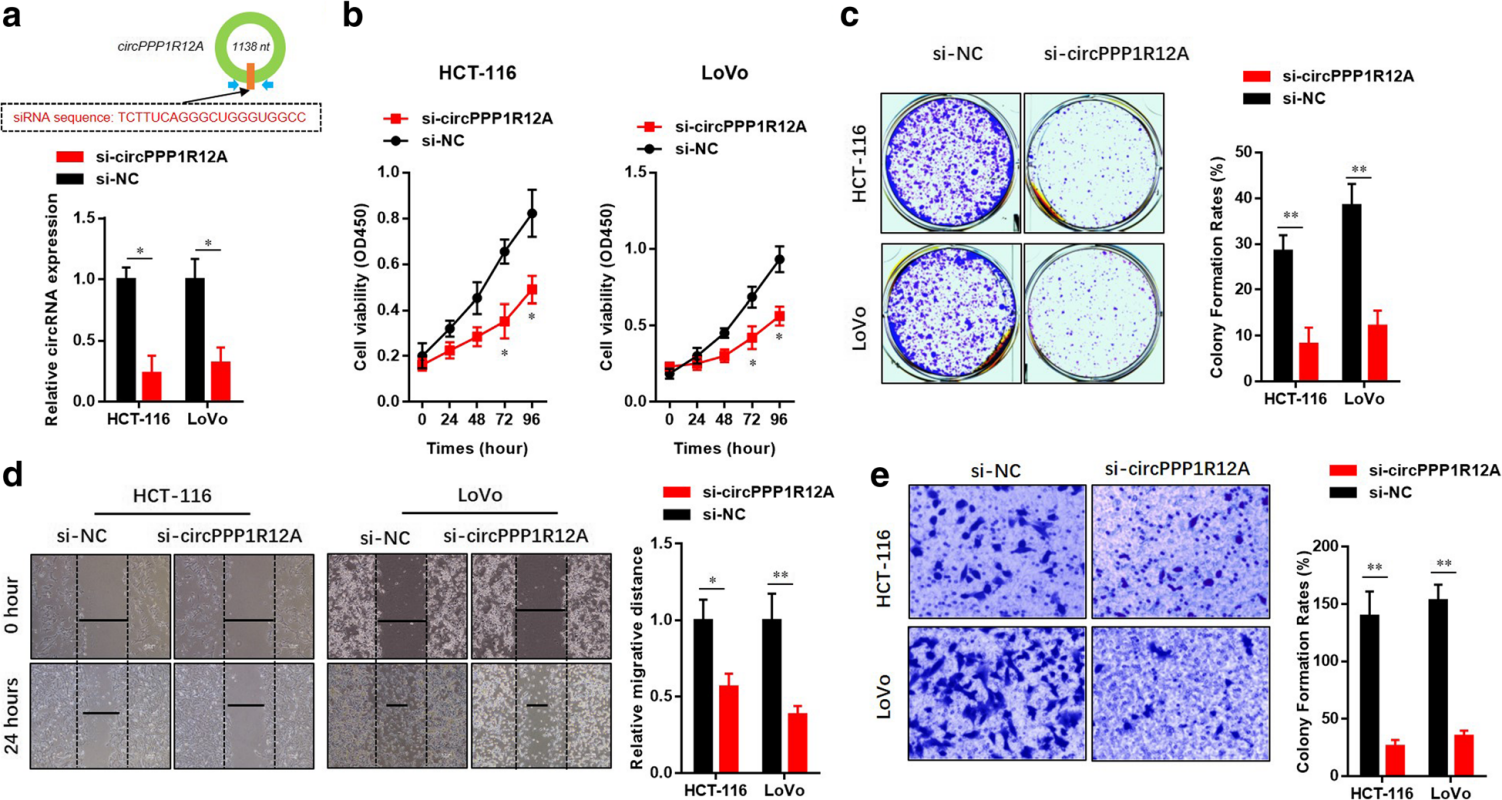
Silencing of circPPP1R12A impairs the cell proliferation, migration and invasion abilities of CC cells. **a** Schematic illustration of siRNA targeting the back-splice junction of circPPP1R12A and real-time PCR confirmation showing circPPP1R12A silencing in HCT-116 and LoVo cells. **b** CircPPP1R12A silencing impaired the proliferation of HCT-116 and LoVo cells shown by CCK8 assay. **c** CircPPP1R12A silencing impaired the proliferation of HCT-116 and LoVo cells shown by colony formation assay. **d** CircPPP1R12A silencing impaired the migration of HCT-116 and LoVo cells shown by wound healing assay. **e** CircPPP1R12A silencing impaired the invasion of HCT-116 and LoVo cells shown by matrigel assay. The data are represented as the means ± SEM; *P < 0.05, **P < 0.01

We next investigated whether silencing of circPPP1R12A affected cell proliferation, migration and invasiveness. The silencing efficiency was confirmed by real-time PCR (Fig. 4 a). As expected, silencing of circPPP1R12A affected the proliferation (Fig. 4 b) and colony forming abilities (Fig. 4 c) of both cell lines. Furthermore, in wound-healing assay and invasion assay, silencing of circPPP1R12A significantly affected the migration (Fig. 4 d) and invasion (Fig. 4 e) abilities of HCT-116 and LoVo cells. Collectively, these results suggested that circPPP1R12A played a critical role in proliferation, migration and invasion of CC cells.

### CircRNA circPPP1R12A encodes a small uncharacterized protein

**Fig. 5:**
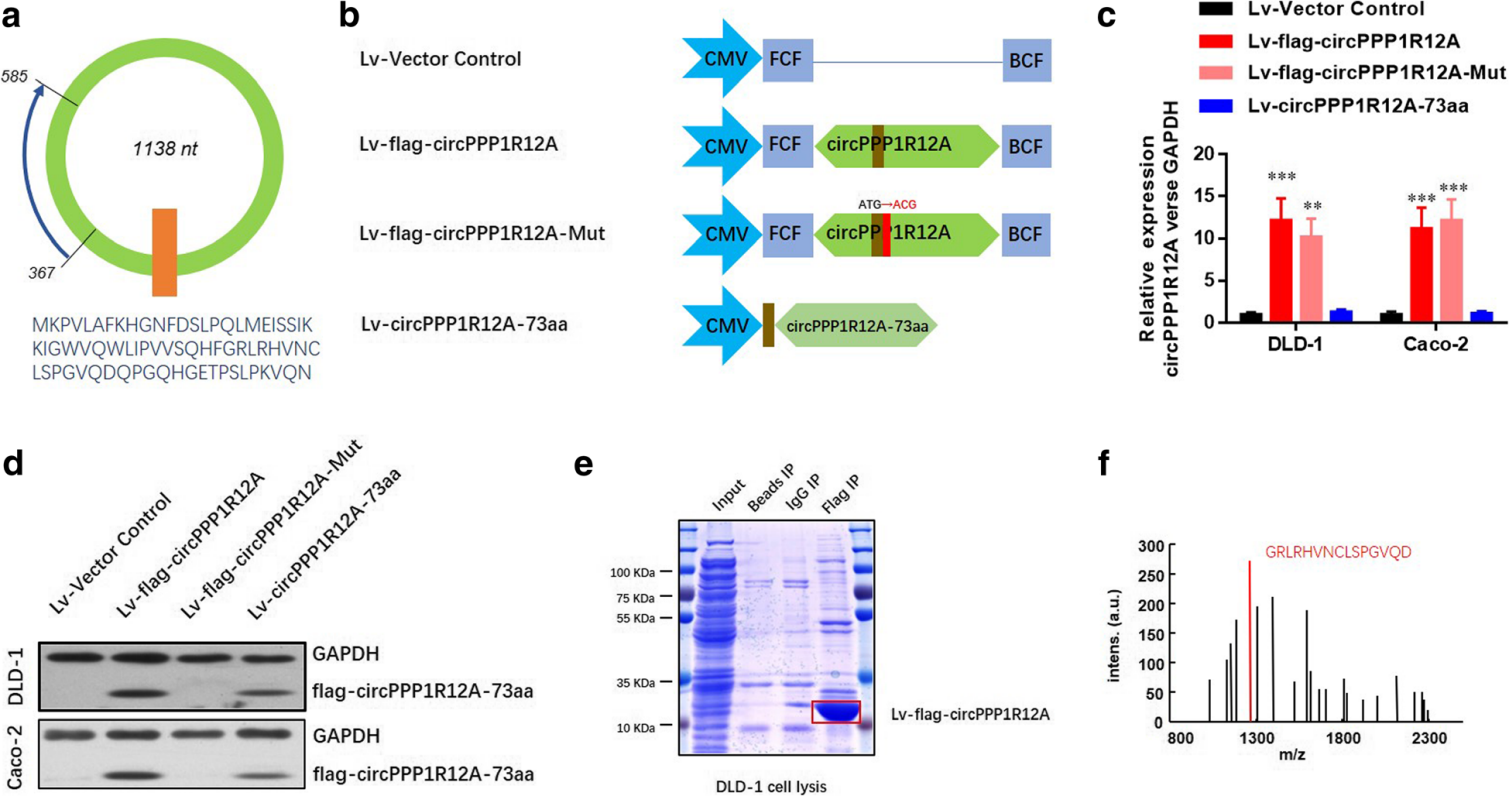
CircRNA circPPP1R12A encodes a small unchartered protein circPPP1R12A-73aa. **a** The 1138-nt circPPP1R12A was potentially translated into a 73-aa (~ 10 kDa) protein (circPPP1R12A-73aa). **b** To establish a detectable PPP1R12A-C expression vector, flag-labeled circPPP1R12A sequence was cloned into a CMV-induced expression vector (Lv-flag-circPPP1R12A). For the circPPP1R12A-73aa deleted circPPP1R12A expression vector, flag-labeled circPPP1R12A sequence with start codon mutant (ATG/ACG) was cloned into a CMV-induced expression vector (Lv-flag-circPPP1R12A-Mut). For the circPPP1R12A-73aa positive control, the flag-labeled circPPP1R12A-73aa sequence was cloned into a CMV-induced expression vector (Lv- circPPP1R12A-73aa). The schematic structure of these vectors was shown. **c** The expression level of circPPP1R12A was analyzed by real-time PCR. **d** The expression level of flag-indicated circPPP1R12A-73aa was detected by Western blotting analysis. **e** The pulldown cell lysates from indicated groups were separated by SDS-PAGE. Protein bands at 10 kDa were manually excised and submitted for identification by LC-MS/MS. **f** LC-MS/MS-identified sequences covered 76% of the aa sequences of circPPP1R12A-73aa. Unique aa sequences of spectrum (GRLRHVNCLSPGVQD) formed by circular junction of circPPP1R12A-73aa are shown. The data are represented as the means ± SEM; *P < 0.05, **P < 0.01, ***P < 0.001

As circRNA circPPP1R12A is originally annotated in circRNA Db database, we found a short 216-nt small ORF with the potential to encode a conserved 73-aa peptide (Fig. 5 a). Especially, in human circPPP1R12A, the circularization created the tandem start codon ‘AUG’, which started the translation in combination with overlapping genetic codes. The ORF of circPPP1R12A was supposed to be translated into a putative 73-aa protein (we termed ‘circPPP1R12A-73aa’), in which the unique aa sequences were formed (Fig. 5 a). To dissociate the role of circPPP1R12A-73aa from circPPP1R12A, we constructed several flag-labeled vectors for circPPP1R12A (Fig. 5 b). Transfection with the Lv-flag-circPPP1R12A vector and Lv-flag-circPPP1R12A-Mut vector both successfully resulted in the overexpression of circPPP1R12A, while transfection with the control vector or Lv-circPPP1R12A-73aa did not (Fig. 5 c). Moreover, the level of flag-labeled circPPP1R12A-73aa protein was elevated after Lv-OE-flag-circPPP1R12A-73aa or Lv-circPPP1R12A-73aa transfection (Fig. 5 d). We further confirmed that circPPP1R12A-73aa was translated from circPPP1R12A by LC-MS/MS, and the unique aa sequence of circPPP1R12A-73aa was identified. More than 76% of the circPPP1R12A-73aa sequences were identified in the LC-MS/MS results from the 10-kDa band (Fig. 5 e). In addition, one unique aa sequence formed by the circPPP1R12A “GRLRHVNCLSPGVQD” was identified (Fig. 5 f). Taken together, circPPP1R12A, which was annotated as a circRNA, actually encoded an uncharacterized protein.

### CircPPP1R12A-73aa, not circPPP1R12A, promotes the proliferation and metastasis abilities of CC in vitro and in vivo

**Fig. 6:**
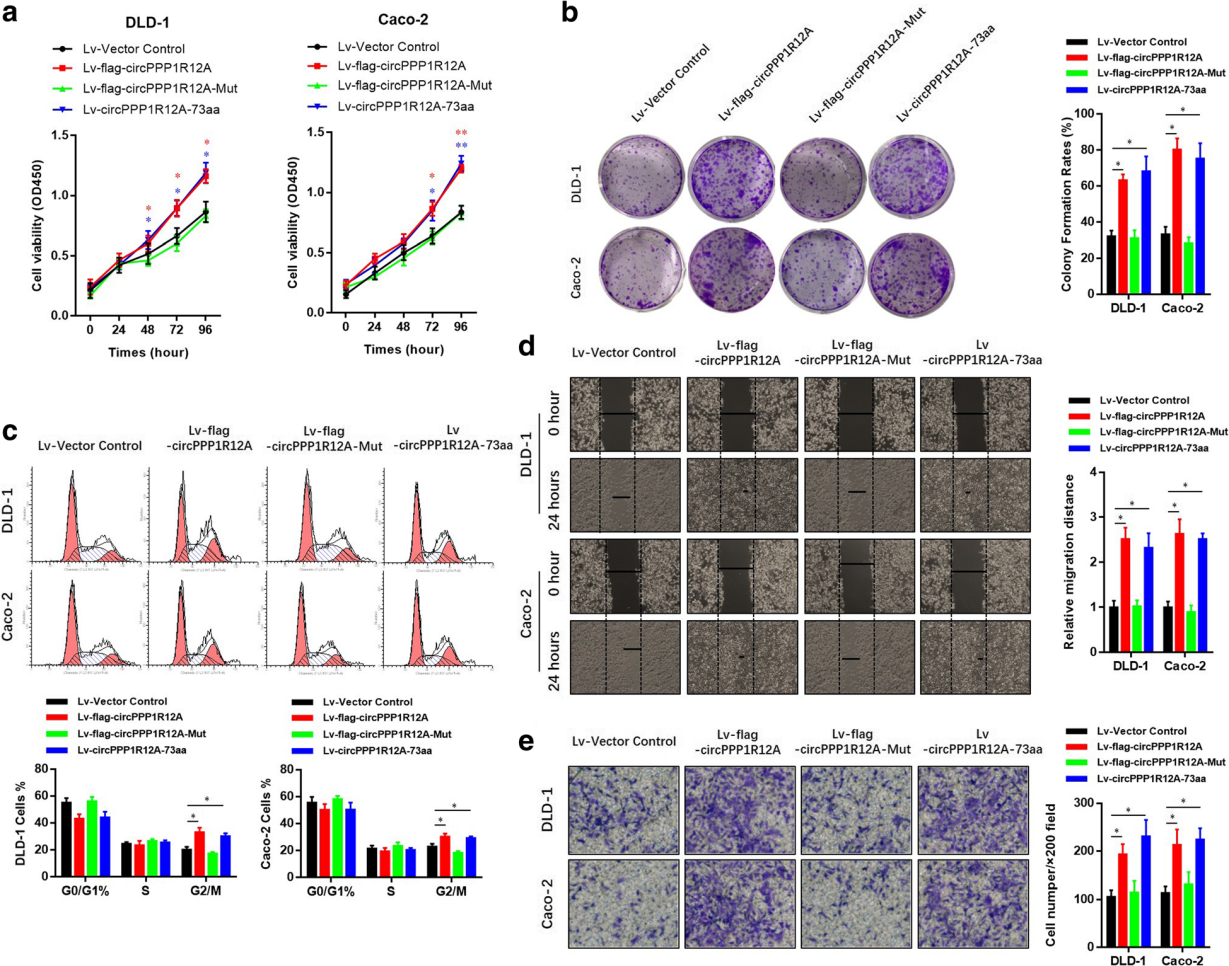
CircPPP1R12A-73aa, not circPPP1R12A, promotes the proliferation ability of CC in vitro. **a** CircPPP1R12A-73aa, not circPPP1R12A promoted the proliferation of HCT-116 and LoVo cells shown by CCK8 assay. **b** CircPPP1R12A-73aa, not circPPP1R12A, promoted the proliferation of HCT-116 and LoVo cells shown colony formation assay. **c** CircPPP1R12A-73aa, not circPPP1R12A, promoted the proliferation of HCT-116 and LoVo cells shown cell cycle. The data are represented as the means ± SEM; *P < 0.05, **P < 0.01. The red * indicated the Lv-flag-circPPP1R12A-WT group, The blue * indicated the Lv-circPPP1R12A-73aa group

To further explore the biological function of circPPP1R12A-73aa, we generated several cell lines with stable transfection of those above-mentioned four vectors (Fig. 5 b). Similar with the previous results (Fig. 3 b), overexpression of wild-type circPPP1R12A with flag labeling resulted in increased proliferation ability, evidenced by CCK8 (Fig. 6 a), colony formation (Fig. 6 b) and cell cycle (Fig. 6 c) assays in DLD-1 and Caco-2 cells. Indeed, only overexpression of circPPP1R12A-73aa increased the proliferation ability too. However, overexpression of mutant circPPP1R12A with flag labeling, which was unable to translate circPPP1R12A-73aa, failed to increase the proliferation ability of DLD-1 and Caco-2 cells. For the motility of CC cells, overexpression of circPPP1R12A-73aa increased the migration (Fig. 6 d) and invasion (Fig. 6 e) abilities too, while overexpression of mutant circPPP1R12A failed to increase the migration and invasion abilities of DLD-1 and Caco-2 cells. These data suggested that circPPP1R12A did not regulate the cell proliferation of CC cells without its encoding protein circPPP1R12A-73aa.

**Fig. 7:**
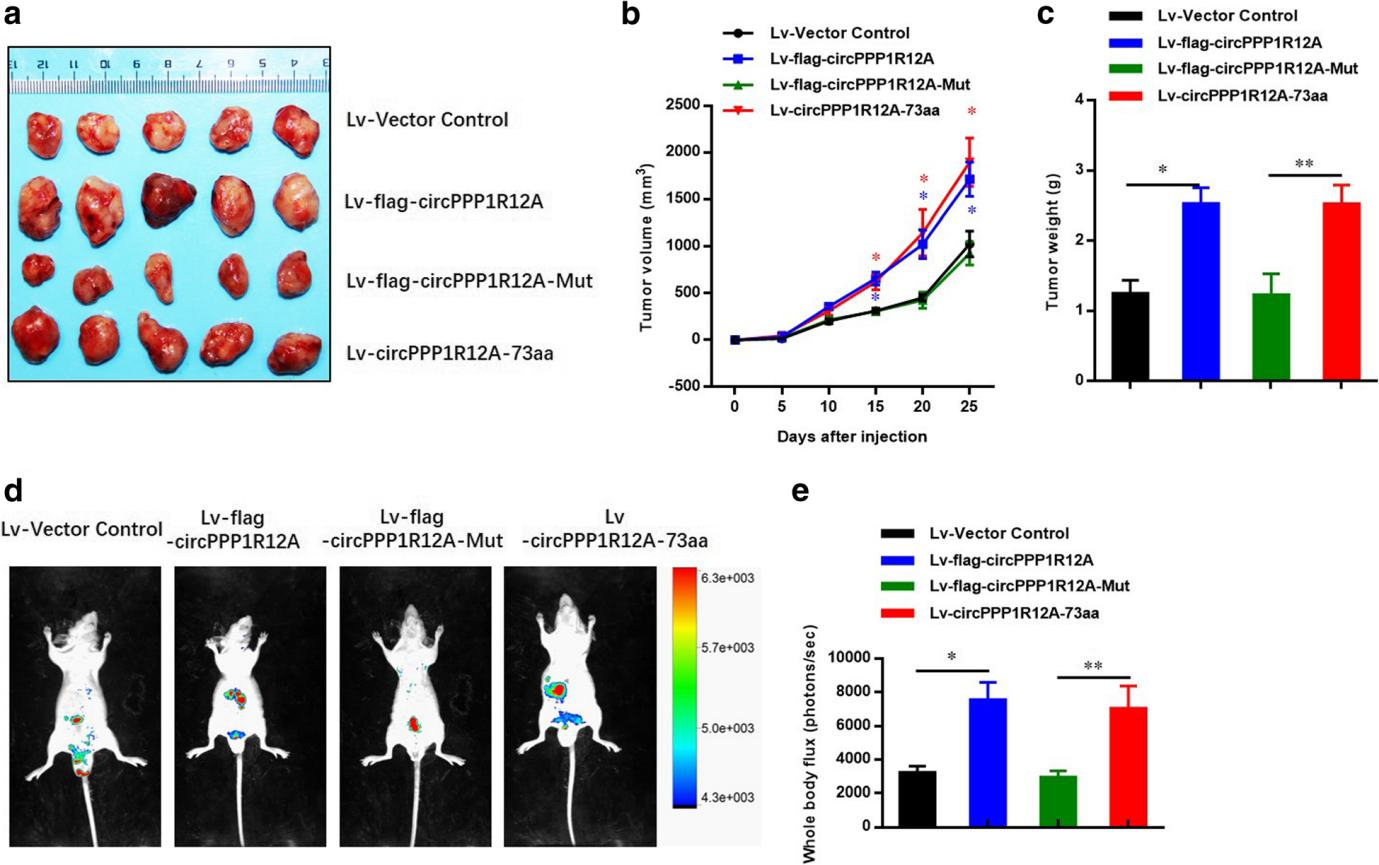
CircPPP1R12A-73aa, not circPPP1R12A, promotes the in vivo tumorigenicity ability of CC using nude mice xenografts. **a** Representative nude mice xenograft formed by the indicated cells. **b** Statistical analysis of xenograft tumor growth formed by the indicated cells. **c** Tumor weights of the indicated cells. d Bioluminescent Imaging (BLI) of mice as indicated (Red arrow indicates the metastatic site in BLI of mice). The images were representative of the data. Counts are photons detected. Images were captured with a 5 min exposure. Whole body flux (photons/sec) quantification of mice injected with different 1 × 10^5^ DLD-1 cells at day 25 (*n* = 5). The data are represented as the means ± SEM; *P < 0.05, **P < 0.01, ***P < 0.001. The red * indicated the Lv-flag-circPPP1R12A-WT group, The blue * indicated the Lv-circPPP1R12A-73aa group

Next, we further investigated the role of circPPP1R12A-73aa in growth of CC cells in vivo using a xenograft tumor model. Figure 7 shows that the tumors generated from cells transfected with Lv-flag-circPPP1R12A and Lv-circPPP1R12A-73aa had a bigger size (Fig. 7 a) and larger weight (Fig. 7 b) compared with those generated from vector control cells. Similar with the in vitro data, the tumors generated from cells transfected with Lv-flag-circPPP1R12A-mut had a smaller size (Fig. 7 a) and lower weight (Fig. 7 c). As the liver metastasis of CC is the most common metastasis in vivo, we then assessed the effects of circPPP1R12A-73aa on tumor liver metastasis by injecting four stably transfected DLD-1 cells (Fig. 5 b) into the tail veins of nude mice. Subsequently, in vivo bioluminescent imaging (BLI) of mice showed that overexpression of circPPP1R12A-WT and circPPP1R12A-73aa significantly changed the detectable metastasis (red arrow indicates metastasis) in the liver compared with the controls and the circPPP1R12A-Mut group (Fig. 7 d and e).

Collectively, these data showed that circPPP1R12A-73aa encoded by circPPP1R12A, not circPPP1R12A, promoted the cell proliferation and metastasis of CC cells.

### circPPP1R12A-73aa promotes the proliferation, migration and invasion abilities of CC via activating hippo-YAP signaling pathway

**Fig. 8:**
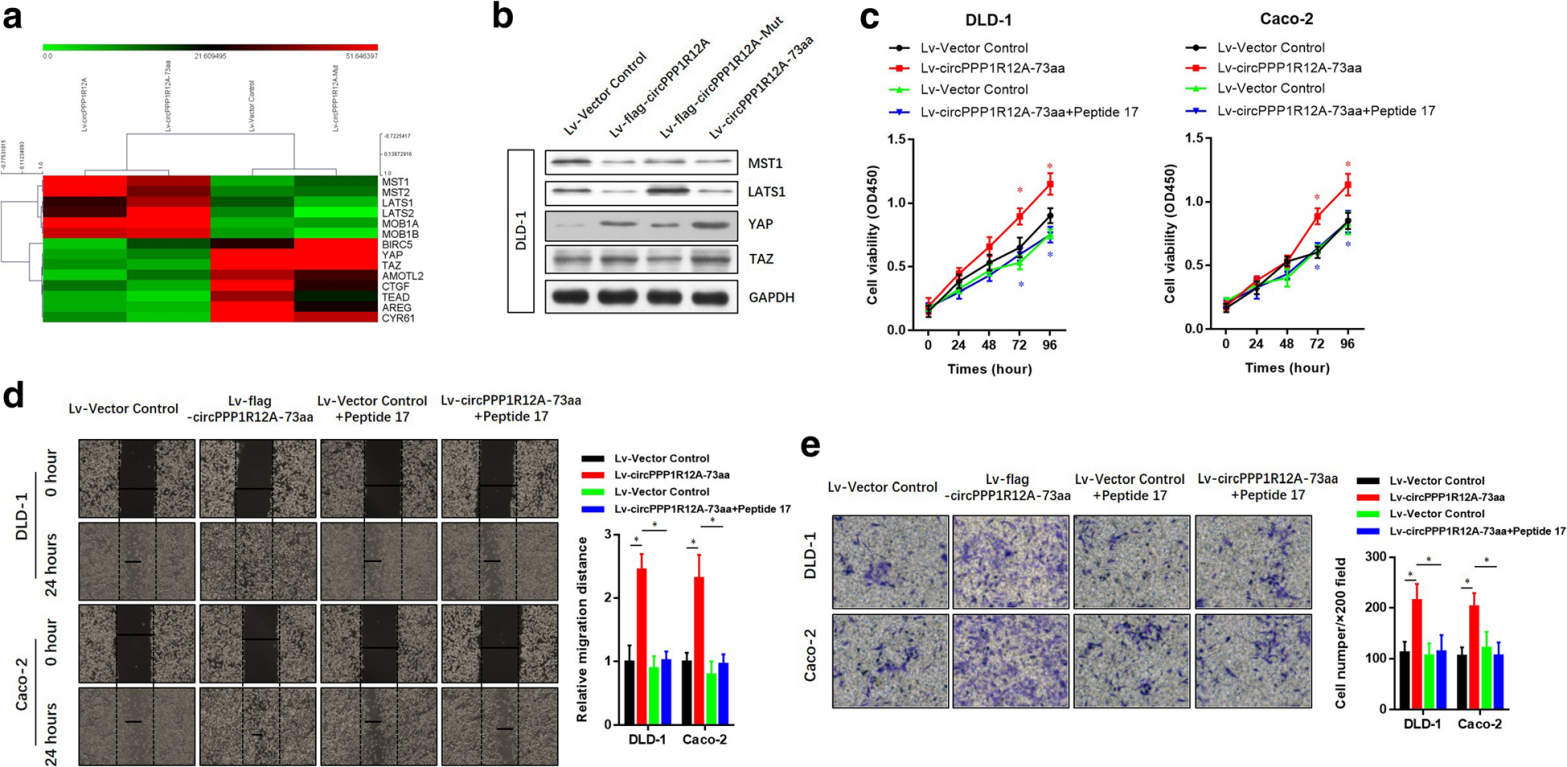
circPPP1R12A-73aa promotes the proliferation, migration and invasion abilities of CC via activating Hippo-YAP signaling pathway. **a** heatmap, generated from the RNA-seq analysis, showing differential gene expression between the indicated four groups. **b** Western Blot was used to evaluate the expression level of MST1, LAST1, YAP and TAZ. **c** The cell viability was analyzed by CCK8 assay to evaluate the YAP inhibition on the circPPP1R12A-73aa promoted proliferation. **d** The migration ability was analyzed by wound healing assay to evaluate the YAP inhibition on the circPPP1R12A-73aa promoted proliferation. **e** The invasion ability was analyzed by transwell assay to evaluate the YAP inhibition on the circPPP1R12A-73aa promoted proliferation. The data are represented as the means ± SEM; *P < 0.05. The red * indicated the Lv-flag-circPPP1R12A-WT group, The blue * indicated the Lv-circPPP1R12A-73aa group

**Fig. 9:**
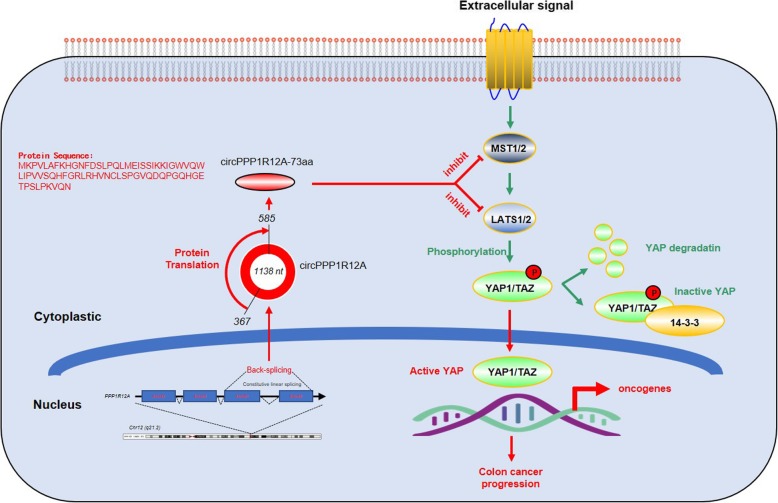
A working model of circPPP1R12A-73aa on promoting tumor pathogenesis and metastasis of CC via activating Hippo-YAP signaling pathway

RNA-Seq analyses were carried out to identify the possible signaling pathways, by which circPPP1R12A-73aa promoted the proliferation, migration and invasion. RNA from the four stably transfected DLD cells (Fig. 5 b) was isolated and analyzed. Differentially expressed genes in the four groups were analyzed and enrichened in KEGG pathway database. To elucidate the regulatory mechanism of the signaling influenced by circPPP1R12A-73aa, we focused on the sub-category of signal transduction pathway from the KEGG pathway database. The pathways in this category were ranked, and Fig. 8 a lists the top 10 pathways. As the most enriched pathway, the Hippo-YAP signaling pathway was further proved to be affected by circPPP1R12A-73aa. We next conducted Western blotting analysis to evaluate the dysregulated genes in Hippo-YAP signaling (Fig. 8 b). YAP1 is a transcriptional co-activator in the Hippo signaling pathway, and YAP1-induced transcriptional responses are essential in proliferation and metastasis of cancer cells. To illustrate whether YAP1 activation was critical for the circPPP1R12A-73aa-induced growth and metastasis of cancer cells, we then treated the circPPP1R12A-73aa-overexpressing DLD-1 and Caco-2 CC cells with YAP1 specific inhibitor Peptide 17. The results revealed that the Peptide 17 significantly alleviated the promotive effect of circPPP1R12A-73aa overexpression on the proliferation (Fig. 8 c), migration (Fig. 8 d) and invasion (Fig. 8 e) abilities of CC cells. Therefore, we confirmed that circPPP1R12A-73aa promoted the proliferation, migration and invasion abilities of CC via activating Hippo-YAP signaling pathway (Fig. 9).

## Discussion

Recently, human genome has been largely transcribed, and non-coding transcripts are found to be abundant in the human transcriptome [5, 23]. As a type of ncRNAs, circRNAs have recently attracted wide attention [9, 11]. The broad expression pattern of circRNAs strongly elucidates their roles in regulation of carcinogenesis [13, 24–26]. Recent study has revealed a tumor suppressive protein encoded by the circular form of the SHPRH gene in glioma tumorigenesis [16]. However, it remains unknown whether there are protein-coding circRNAs involved in CC tumorigenesis.

In the present study, we screened the expression profiles of circRNAs between CC tissues and matched adjacent non-tumor tissues, and identified a most elevated circRNA circPPP1R12A in primary cancer tissues and cancer cell lines. We further provided the evidence that the circRNA circPPP1R12A encoded a conserved 73-aa small peptide, circPPP1R12A-73aa. Moreover, circPPP1R12A-73aa, not circPPP1R12A circRNA itself, promoted the growth and metastasis of CC cells in vitro and in vivo. On the other hand, PPP1R12A, also called the myosin-binding subunit of myosin phosphatase, is one of the subunits of myosin phosphatase, which is frequently expressed at a low level in human cancers. PPP1R12A is mainly involved in the RhoA/ROCK signaling pathway, which participates in the regulation of adhesion, movement, proliferation, differentiation and apoptosis of cells. However, the expression of PPP1R12A was not significantly different between CC tissues and paired adjacent tissues. In addition, we found that PPP1R12A was expressed at a low level in CC tissues. More studies on alternative splicing during the transcription of PPP1R12A might be carried out to illustrate the regulatory mechanism.

After the earliest characterized circRNA, the sex-determining region of ChrY (Sry), is found [27], cerebellar degeneration-related protein 1 (CDR1as/ciRS-7) has been further demonstrated as an antisense transcript to sponge miR-7 and suppress miR-7 activity [13, 28, 29]. Several cancer-derived circRNAs, including circHIPK3 [18], circMTO1 [12], circCCDC66 [23] and circPVT1 [30], have been reported to be involved in multiple cancers. The functions for circRNAs as well as their underlying mechanisms in carcinogenesis and cancer progression remain largely unexplored. The circRNAs mainly act as ceRNAs, forming circRNA-miRNA-mRNA axis. To the best of our knowledge, circPPP1R12A was the first eukaryotic circRNA encoding small protein in CC, suggesting the complexity of carcinogenesis. The translated product of circPPP1R12A shared most of its aa sequence with that of PPP1R12A, except for the unique aa sequence in the ORF located in the C-terminus. However, the underlying mechanisms by which PPP1R12A-circPPP1R12A-73aa regulated the motility of CC cells needed to be further illustrated.

## Conclusions

Collectively, we found an elevated circRNA circPPP1R12A in the cytoplasm of CC tissues and cells. In addition, evidence indicated that the circRNA circPPP1R12A encoded a conserved 73-aa small peptide, PPP1R12A-C. The PPP1R12A-C peptide, not circPPP1R12A circRNA itself, promoted the growth of CC cells in vitro and in vivo. In addition, circPPP1R12A-73aa promoted the proliferation, migration and invasion abilities of CC cells via activating Hippo-YAP signaling pathway. Taken together, our study intuitively illustrated the coding potential of circRNAs in the progression of CC. Moreover, our findings might provide valuable insights into the development of potential therapeutic targets for CC.

## Additional files

Additional file 1: **Table S1.** Detailed information of the differentially expressed circRNAs. (XLS 66 kb)

Additional file 2: **Figure S1.** PPP1R12A expression in CC tissues and cells. **a** The expression level of PPP1R12A in CC and matched non-tumor tissue samples from 20 patients was analyzed by real-time PCR. **b** The expression level of PPP1R12A in a series of cultured CC cell lines (HT-29, HCT-116, SW480, SW620, LoVo, SW48, DLD-1, Caco2 and HCT-15) was analyzed by real-time PCR. (JPG 50 kb)

Additional file 3: **Figure S2.** Circular RNA circPPP1R12A promotes the cell proliferation, but not migration and invasion abilities of HCT-116 and LoVo colon cancer cells. **a** circPPP1R12A promotes the proliferation of HCT-116 and LoVo cells shown by CCK8 assay. **b** circPPP1R12A promotes the proliferation of HCT-116 and LoVo cells shown by colony formation assay. **c** circPPP1R12A did not affect the migration of HCT-116 and LoVo cells shown by wound healing assay. **d** circPPP1R12A did not affect the invasion of HCT-116 and LoVo cells shown by matrial assay. The data are represented as the means ± SEM; *P < 0.05, *N.S*, not significant. (JPG 402 kb)

## Abbreviations

circRNAs: Circular RNAs
FISH: fluorescence in situ hybridization
ISH: in situ hybridization
ORF: open reading frame
