# Correction to: A novel protein encoded by a circular RNA circPPP1R12A promotes tumor pathogenesis and metastasis of colon cancer via Hippo-YAP signaling

**DOI:** 10.1186/s12943-021-01337-3

**Published:** 2021-02-25

**Authors:** Xiao Zheng, Lujun Chen, You Zhou, Qi Wang, Zhuojun Zheng, Bin Xu, Chen Wu, Qi Zhou, Wenwei Hu, Changping Wu, Jingting Jiang

**Affiliations:** 1 Department of Tumor Biological Treatment, the Third Affiliated Hospital of Soochow University, Changzhou, 213003 People’s Republic of China; 2 Jiangsu Engineering Research Center for Tumor Immunotherapy, Changzhou, 213003 People’s Republic of China; 3 Institute of Cell Therapy, Soochow University, Changzhou, 213003 People’s Republic of China; 4 Department of Hematology, the Third Affiliated Hospital of Soochow University, Changzhou, 213003 People’s Republic of China; 5 Department of Oncology, the Third Affiliated Hospital of Soochow University, Changzhou, 213003 People’s Republic of China

**Correction to: Mol Cancer 18, 47 (2019)**

**https://doi.org/10.1186/s12943-019-1010-6**

Following the publication of the original article [1], the authors identified some minor errors in image-typesetting in Figs. 2 and 7; specifically the DAPI image panels in Fig. 2d, and Fig. 7a. The corrected images have been provided here. The correction has no impact on the results or conclusions of the study.

**Fig. 2:**
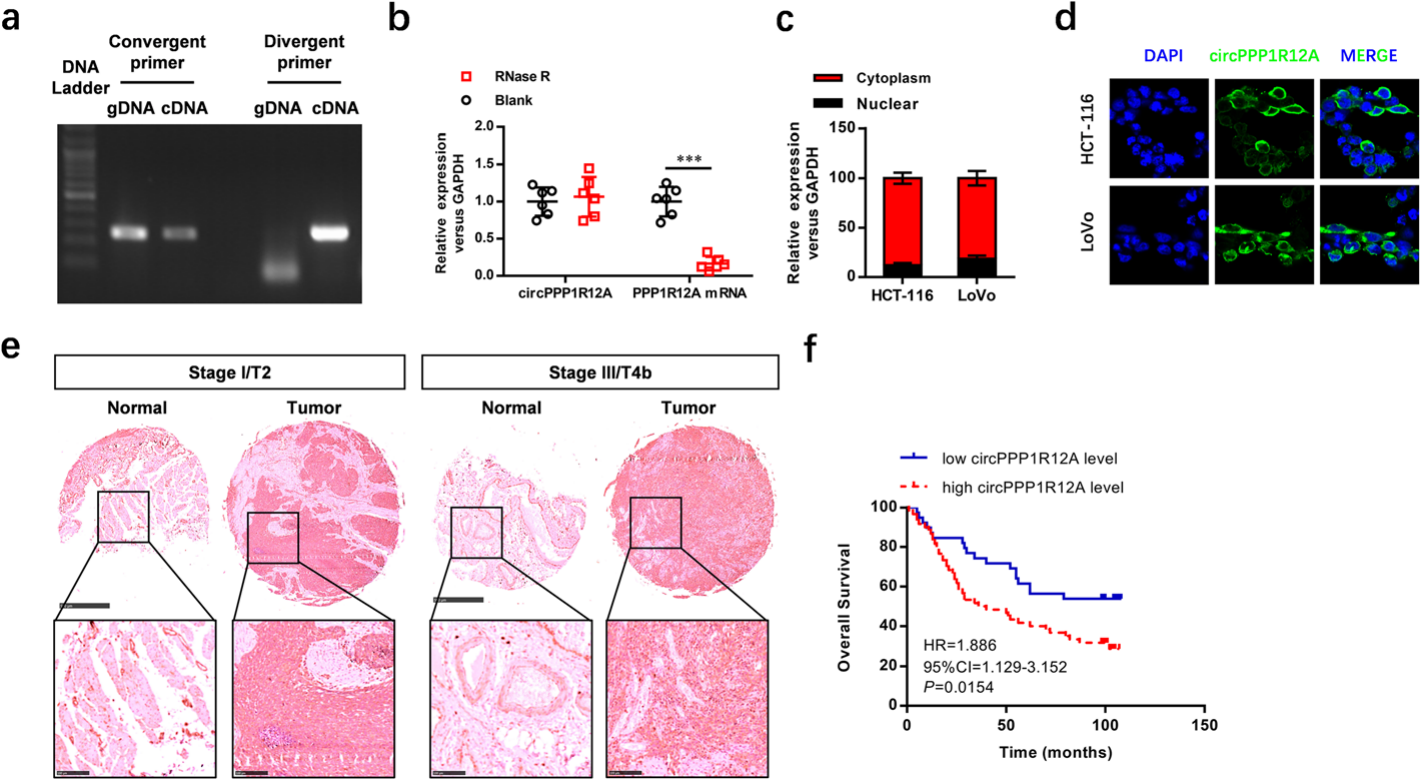
Characterization the existence and subcellular distribution of circPPP1R12A in CC cells and tissues. **a** The divergent primers detected circPPP1R12A in cDNA but not in gDNA. **b** Real-time PCR analysis of circPPP1R12A and linear PPP1R12A mRNA after treatment with RNase R in HCT-116 cells showed that circPPP1R12A was resistant to RNase R treatment. The sub-cellular distribution of circPPP1R12A was mostly present in the cytoplasm by the nuclear mass separation assay (**c**) and FISH (**d**). **e** The level of circPPP1R12A was analyzed by in situ hybridization on CC tissue microarray, showing that circPPP1R12A was up-regulated in CC tissues compared with normal tissues, and such up-regulation was positively correlated with larger tumors and a higher TNM stage. **f** Kaplan–Meier analysis of the correlation between circPPP1R12A expression and overall survival showed that patients with higher levels of circPPP1R12A had a significantly shorter overall survival. ***P < 0.001

**Fig. 7:**
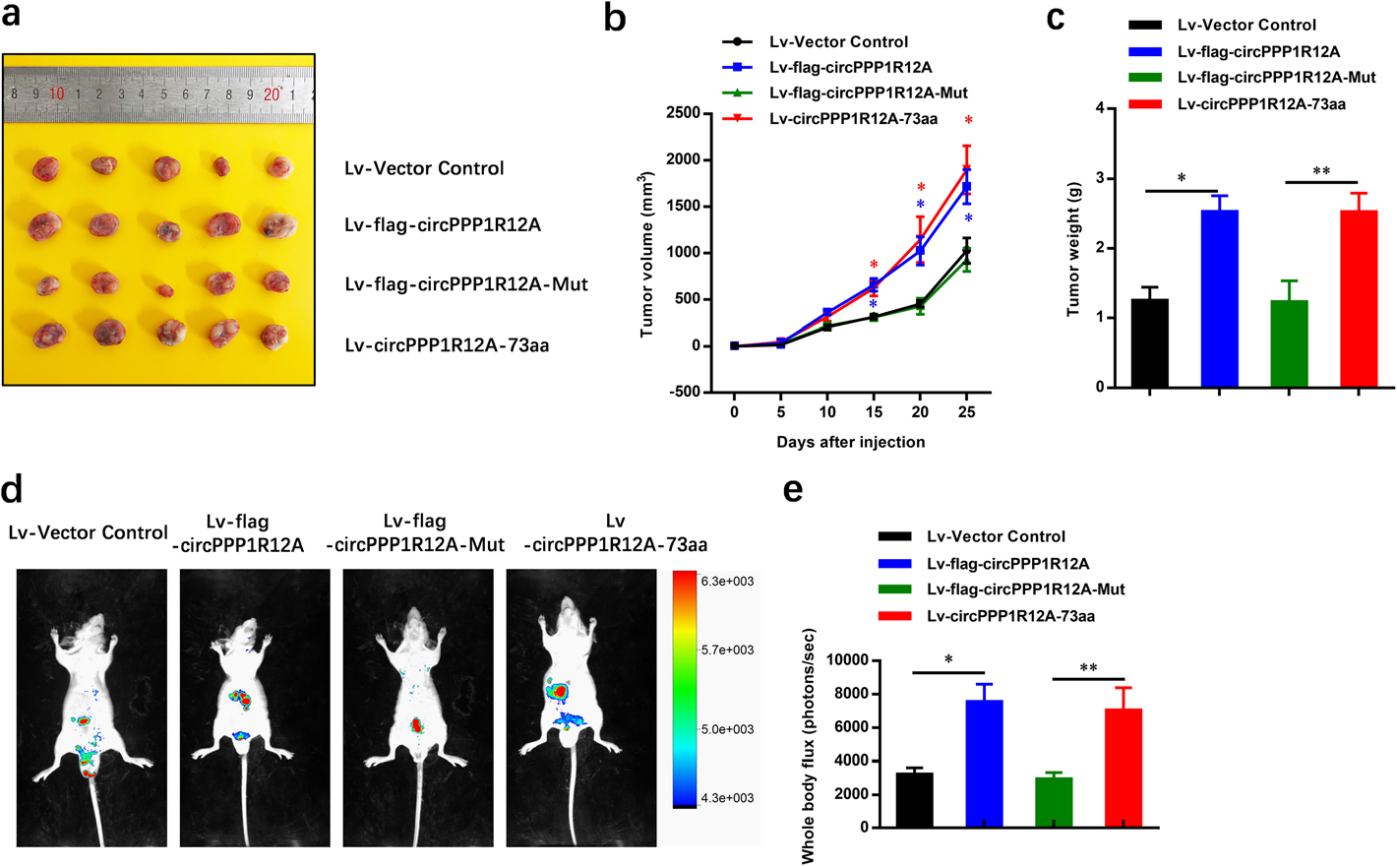
CircPPP1R12A-73aa, not circPPP1R12A, promotes the in vivo tumorigenicity ability of CC using nude mice xenografts. **a** Representative nude mice xenograft formed by the indicated cells. **b** Statistical analysis of xenograft tumor growth formed by the indicated cells. **c** Tumor weights of the indicated cells. d Bioluminescent Imaging (BLI) of mice as indicated (Red arrow indicates the metastatic site in BLI of mice). The images were representative of the data. Counts are photons detected. Images were captured with a 5 min exposure. Whole body flux (photons/sec) quantification of mice injected with different 1 × 10^5^ DLD-1 cells at day 25 (*n* = 5). The data are represented as the means ± SEM; *P < 0.05, **P < 0.01, ***P < 0.001. The red * indicated the Lv-flag-circPPP1R12A-WT group, The blue * indicated the Lv-circPPP1R12A-73aa group
