# Supplementary figures and images for: A novel protein encoded by a circular RNA circPPP1R12A promotes tumor pathogenesis and metastasis of colon cancer via Hippo-YAP signaling
Source: Mol Cancer. 2019 Mar 29;18:47. doi: 10.1186/s12943-019-1010-6 (PMC6440158; doi:10.1186/s12943-019-1010-6)

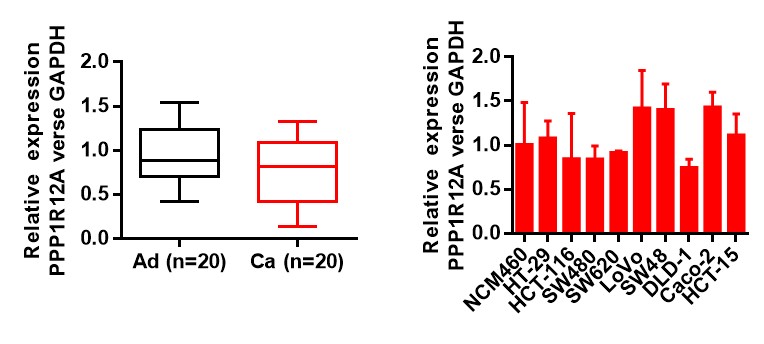

Supplement: Supplementary file 2 — Figure S1. PPP1R12A expression in CC tissues and cells. a The expression level of PPP1R12A in CC and matched non-tumor tissue samples from 20 patients was analyzed by real-time PCR. b The expression level of PPP1R12A in a series of cultured CC cell lines (HT-29, HCT-116, SW480, SW620, LoVo, SW48, DLD-1, Caco2 and HCT-15) was analyzed by real-time PCR. (JPG 50 kb) [file 12943_2019_1010_MOESM2_ESM.jpg]

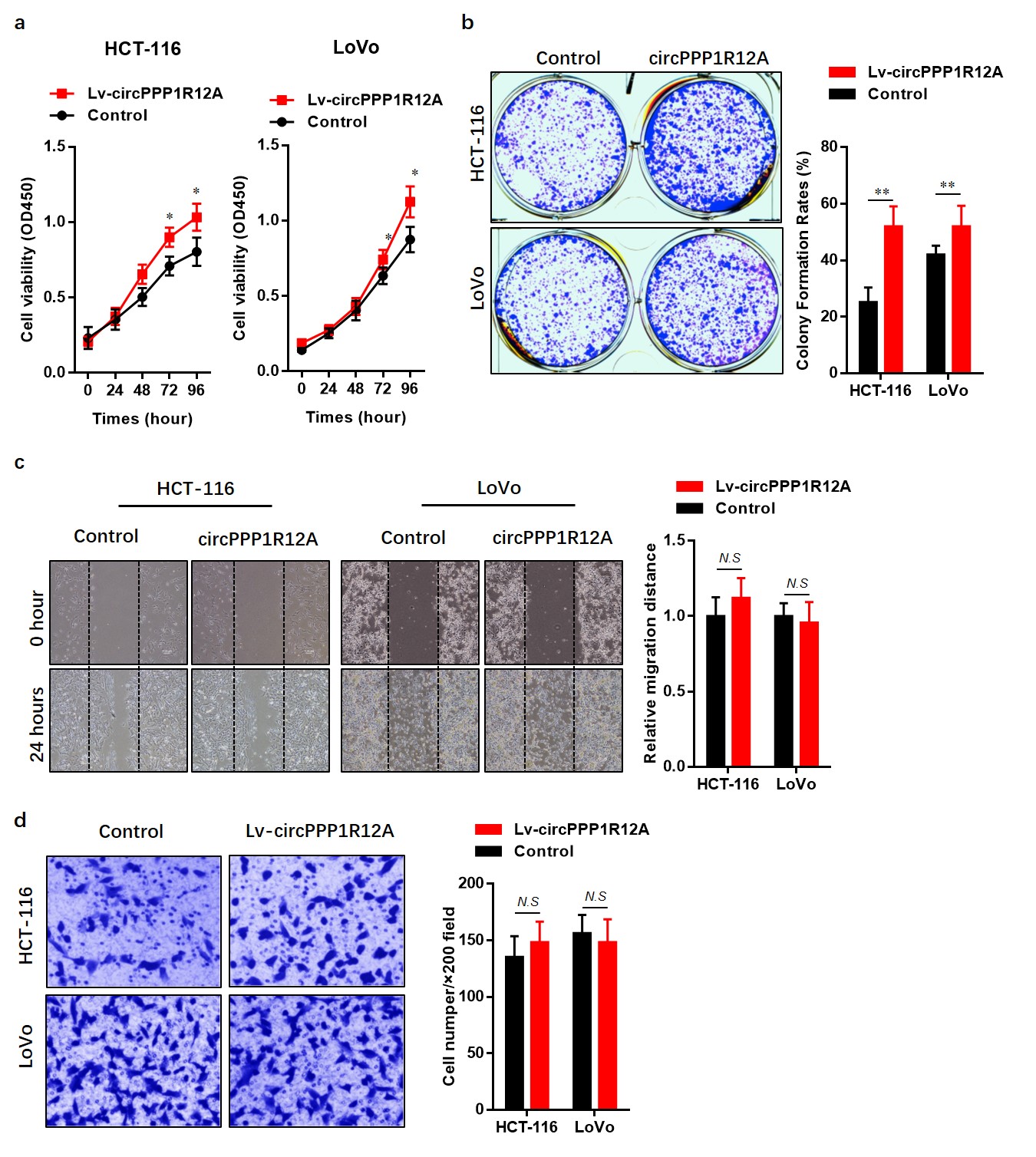

Supplement: Supplementary file 3 — Figure S2. Circular RNA circPPP1R12A promotes the cell proliferation, but not migration and invasion abilities of HCT-116 and LoVo colon cancer cells. a circPPP1R12A promotes the proliferation of HCT-116 and LoVo cells shown by CCK8 assay. b circPPP1R12A promotes the proliferation of HCT-116 and LoVo cells shown by colony formation assay. c circPPP1R12A did not affect the migration of HCT-116 and LoVo cells shown by wound healing assay. d circPPP1R12A did not affect the invasion of HCT-116 and LoVo cells shown by matrial assay. The data are represented as the means ± SEM; *P < 0.05, N.S, not significant. (JPG 402 kb) [file 12943_2019_1010_MOESM3_ESM.jpg]
